# Integrated metabolome and transcriptome analysis of the anthocyanin biosynthetic pathway in relation to color mutation in miniature roses

**DOI:** 10.1186/s12870-021-03063-w

**Published:** 2021-06-04

**Authors:** Jiaojiao Lu, Qing Zhang, Lixin Lang, Chuang Jiang, Xiaofeng Wang, Hongmei Sun

**Affiliations:** 1 Key Laboratory of Protected Horticulture of Education Ministry and Liaoning Province, College of Horticulture, Shenyang Agricultural University, Shenyang, 110866 China; 2 Liaoning Academy of Agricultural Sciences, Shenyang, 110161 China

**Keywords:** Miniature rose, Mutation, Pigmentation, Transcriptome, Anthocyanin biosynthetic pathway

## Abstract

**Background:**

Roses are famous ornamental plants worldwide. Floral coloration is one of the most prominent traits in roses and is mainly regulated through the anthocyanin biosynthetic pathway. In this study, we investigated the key genes and metabolites of the anthocyanin biosynthetic pathway involved in color mutation in miniature roses. A comparative metabolome and transcriptome analysis was carried out on the Neptune King rose and its color mutant, Queen rose, at the blooming stage. Neptune King rose has light pink colored petals while Queen rose has deep pink colored petals.

**Result:**

A total of 190 flavonoid-related metabolites and 38,551 unique genes were identified. The contents of 45 flavonoid-related metabolites, and the expression of 15 genes participating in the flavonoid pathway, varied significantly between the two cultivars. Seven anthocyanins (cyanidin 3-*O*-glucosyl-malonylglucoside, cyanidin *O*-syringic acid, cyanidin 3-*O*-rutinoside, cyanidin 3-*O*-galactoside, cyanidin 3-*O*-glucoside, peonidin 3-*O*-glucoside chloride, and pelargonidin 3-*O*-glucoside) were found to be the major metabolites, with higher abundance in the Queen rose. Thirteen anthocyanin biosynthetic related genes showed an upregulation trend in the mutant flower, which may favor the higher levels of anthocyanins in the mutant. Besides, eight *TRANSPARENT TESTA 12* genes were found upregulated in Queen rose, probably contributing to a high vacuolar sequestration of anthocyanins. Thirty transcription factors, including two MYB and one bHLH, were differentially expressed between the two cultivars.

**Conclusions:**

This study provides important insights into major genes and metabolites of the anthocyanin biosynthetic pathway modulating flower coloration in miniature rose. The results will be conducive for manipulating the anthocyanin pathways in order to engineer novel miniature rose cultivars with specific colors.

**Supplementary Information:**

The online version contains supplementary material available at 10.1186/s12870-021-03063-w.

## Background

Throughout human history, roses have held a high iconic cultural significance. Roses are used as garden ornamental plants and are employed in various industries such as food and cosmetic. There is a wide variation in flower colors of rose cultivars. In ornamental plants, the floral color is the main attribute and is closely related to the distribution of pigment types [1]. Natural pigments such as flavonoids, carotenoids, betalains, and alkaloids are well known in flower color formation [2–4].

Particularly, flavonoids have attracted more attention as important secondary metabolites in plants [5]. Flavonoids are known to play significant roles in various aspects of plant physiological processes, including protection from ultraviolet radiation, pigmentation, and plant defense [6]. Flavonoids are the products of the phenylpropanoid biosynthesis pathway which has been well characterized in *Arabidopsis* and petunia [7, 8]. Several genes such as phenylalanine lyase, cinnamic acid hydroxylase, 4-coumarate-CoA ligase, chalcone synthase, chalcone isomerase, flavonoid 3-hydroxylase, flavonol synthase, dihydroflavonol 4-reductase, and anthocyanidin synthase, participate at different steps of the pathways. Anthocyanins are the end-products of the flavonoid pathway, but they are unstable in the cytoplasm and require further glycosylation. Genes of the UDP-glucosyltransferase and glutathione S-transferase families enter into action [9, 10]. Conserved structural genes have been identified in the flavonoid biosynthetic pathway, but their regulatory mechanisms vary across plant species [5, 7, 8, 11–14].

As the key aesthetic characteristic of roses, flower color has been early investigated by breeders. Up to date, only few flavonoids have been reported in roses. Rose petals are known to contain anthocyanins, such as pelargonidin, cyanidin, and peonidin [15–17]. The presence of 3,5-diglycosyl anthocyanidins in association with 3-glycosylated flavonols confer the pink and red colors of roses [18, 19]. Three genes (*RhGT1*, *RhGT2*, and *RhGT3*) encoding flavonoid 3-glycosyltransferases were found to be involved in the formation of anthocyanin glycosides [20]. Our understanding of flower coloration mechanisms in roses is far to be complete since several structural genes and as well as transcription factors (TF) regulating the anthocyanin pathways are yet to be reported. Moreover, the large variation existing in modern roses could indicate varying molecular mechanisms of color formation among different roses [21, 22]. High-quality genomes of various *Rosa* species are now available and will facilitate fundamental research on these plants [23–25]. Although some genes involved in flower coloration in roses have been cloned, the lack of mutants for comparative studies makes it impossible to clearly understand the regulation of anthocyanin pathways [26–38].

Understanding the genetic and molecular mechanisms that modify coloration will not only answer a fascinating query involving fundamental rose biology but will also allow the manipulation of rose quality. By integrating metabolome and transcriptome analyses, researchers have identified key metabolites and genes (structural or TF) regulating color formation in plants. In this study, we used a miniature rose cultivar (Neptune King) and its natural color mutant (Queen) and investigated the molecular changes affecting anthocyanin composition in their petals.

## Results

### Metabolome profiling of petal samples

**Fig. 1:**
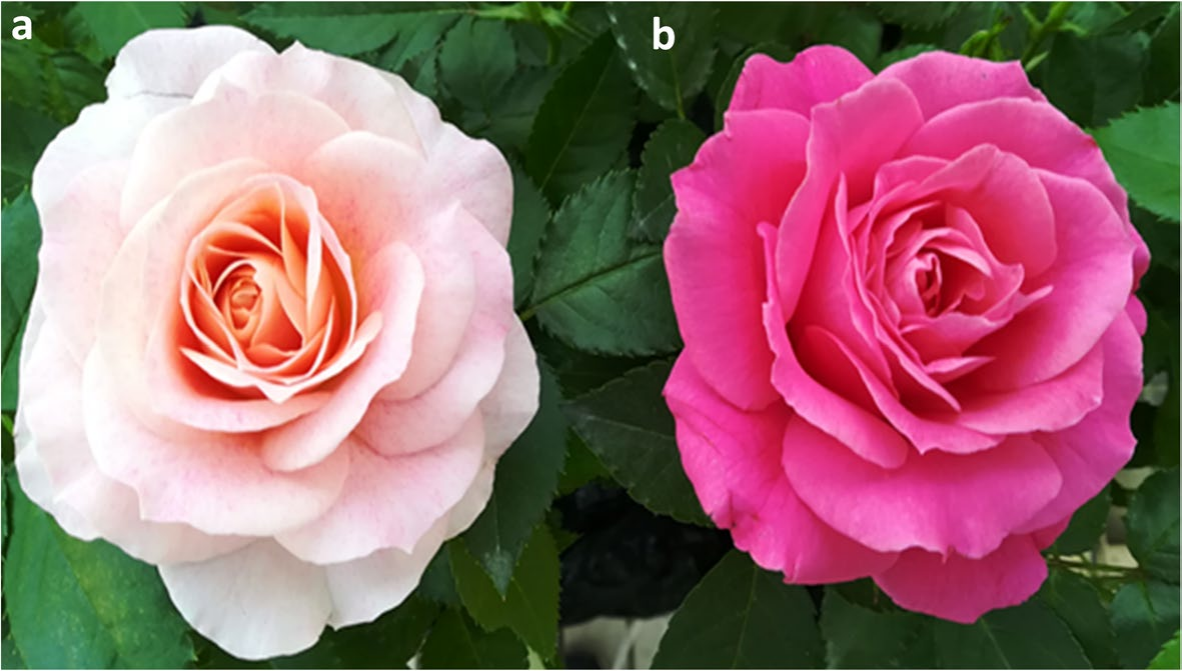
Phenotypes of: a the wild type of miniature rose cultivar Neptune King (H); b Its natural mutant Queen (S)

**Fig. 2:**
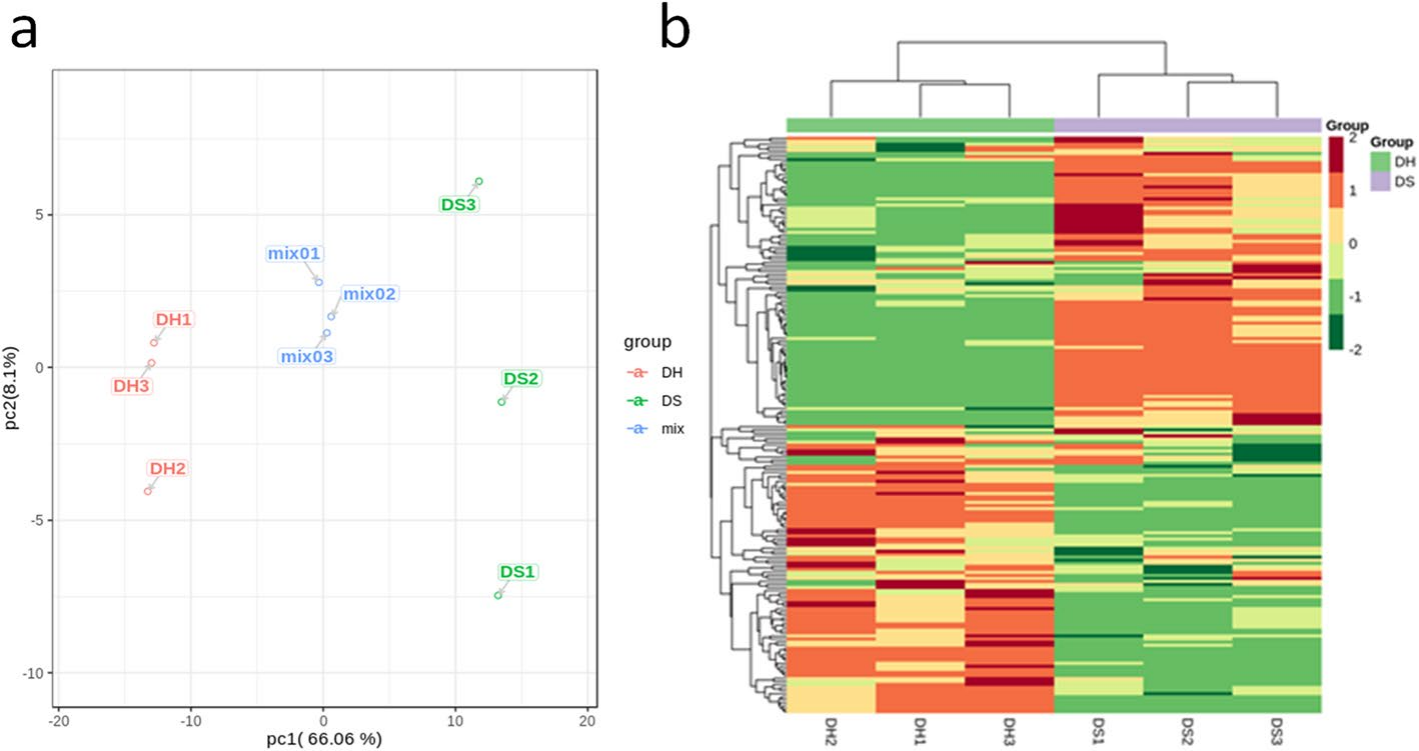
**a** Principal component analysis based on FPKM data; **b** Clustered heatmap based on the 190 metabolites between the flower samples. The color scale (from red to green) represents normalized metabolite contents using the row Z-score. DH, Neptune King metabolites; DS, Queen metabolites

Fresh petals were collected from the miniature rose cultivar Neptune King (H) having light pink color and its natural color mutant Queen (S) displaying deep pink color (Fig. 1a, b). To compare the flavonoid content, petal samples were analyzed using UPLC-MS/MS. The flavonoid profiles of H and S flowers showed marked differences (Figures S1 and S2, Supplementary Materials). Using a local metabolite database, a total of 190 flavonoid-related metabolites were identified, including 18 anthocyanins, 61 flavonols, 8 dihydroflavones, 3 dihydroflavonols, 12 flavonoid carbonosides, 7 isoflavones, 67 other flavonoids, 11 proanthocyanidins, 1 chalcone, and 2 tannins (Table S1, Supplementary Materials). We used the metabolite quantification data to construct a hierarchical clustering heatmap. The results showed a close relationship between biological replicates, a sign of high quality metabolome quantification (Fig. 2a, b). Moreover, it could be observed a clear separation between H and S petal samples, indicating distinct flavonoid profiles in H and S samples.

Differentially accumulated metabolites (DAMs) between H and S petal samples were determined based on the variable importance in projection ≥ 1 and a fold change ≥ 2 or ≤ 0.5. There were 45 metabolites whose accumulation was significantly different between the compared samples, and these differentially accumulated flavonoids (DAFs) were mainly anthocyanins, other flavonoids, procyanidins, and flavonols (Table 1). In particular, seven anthocyanins, namely, cyanidin 3-*O*-glucosyl-malonylglucoside, cyanidin *O*-syringic acid, cyanidin 3-*O*-rutinoside, cyanidin 3-*O*-galactoside, cyanidin 3-*O*-glucoside, peonidin 3-*O*-glucoside chloride, and pelargonidin 3-*O*-glucoside displayed significantly higher content in the S cultivar than in the H cultivar. The quantity of cyanidin *O*-syringic acid was high in the S cultivar but undetectable in the H cultivar. Additionally, the content of cyanidin 3-*O*-glucosyl-malonylglucoside was down-accumulated in the H cultivar compared to the S cultivar. The results indicate that these anthocyanins may play a crucial role for deep pink color formation in S flowers. Thus, the high accumulation of peonidin, pelargonidin, and cyanidin derivatives in the S cultivar may have led to its color-related changes.

**Table 1:** Differentially accumulated flavonoids in H and S petal samples

Group	Metabolite	Content ^a^		VIP ^b^	Fold change ^b^
		**H**	**S**		
Anthocyanins	Peonidin 3-*O*-glucoside chloride	6.91E + 05	6.93E + 06	1.50	10.1
	Pelargonidin 3-*O*-glucoside	1.43E + 07	4.40E + 07	1.06	3.19
	Cyanidin 3-*O*-glucoside	1.80E + 05	1.42E + 06	1.43	8.13
	Cyanidin *O*-syringic acid	9.00E + 00 ^c^	1.63E + 05	3.09	1820
	Cyanidin 3-*O*-rutinoside	6.29E + 05	7.75E + 06	1.56	12.4
	Cyanidin 3-*O*-galactoside	9.80E + 04	1.08E + 06	1.53	11.0
	Cyanidin- 3-O-glucosyl-malonylglucoside	5.13E + 04	6.45E + 03	1.45	0.112
Procyanidins	Procyanidin C2	2.59E + 06	8.37E + 05	1.06	0.317
	Procyanidin C1	1.27E + 05	2.15E + 04	1.32	0.165
	Gallocatechin-catechin	3.34E + 04	7.21E + 03	1.16	0.248
	Epicatechin-epiafzelechin	1.49E + 06	1.76E + 05	1.45	0.116
	Catechin-catechin-catechin	2.09E + 05	3.84E + 04	1.25	0.196
	Procyanidin A2	9.00E + 00 ^c^	2.04E + 05	3.12	2310
Other flavonoids	Kaempferol 3-*O*-β-D-sophoroside	1.43E + 05	4.63E + 05	1.02	3.01
	Quercetin- 3-*O*-(6′′-*trans*-*p*-coumaroyl)-β-D-glucopyranoside	1.68E + 05	6.18E + 05	1.07	3.36
	Kaempferol- 3-*O*-(6′′-*trans*-*p*-coumaroyl)-β-D-glucopyranoside	5.44E + 04	4.00E + 05	1.36	6.83
	Quercetin- 3-*O*-(2′′-*trans*-*p*-coumaroyl)-β-D-glucopyranoside	1.15E + 05	4.54E + 05	1.10	3.58
	Kaempferol- 3-*O*-(2′′-*trans*-*p*-coumaroyl)-β-D-glucopyranoside	6.15E + 04	3.01E + 05	1.22	4.69
	3-*O*-(2′′-Cinnamoyl)-β-D-glucoside	3.60E + 05	1.42E + 06	1.12	3.71
	Quercetin- 7-*O*-malonylhexosyl-hexoside	3.84E + 03	1.04E + 04	1.04	3.22
	5,2′-Dihydroxy-7,8-dimethoxyflavone	9.00E + 00 ^c^	1.31E + 04	2.63	1300
	Apigenin	9.00E + 00 ^c^	1.05E + 04	2.61	1110
	Chrysoeriol *O*-hexosyl-*O*-rutinoside	9.00E + 00 ^c^	2.12E + 04	2.73	2200
	Acacetin-7-*O*-galactoside	1.10E + 05	3.44E + 05	1.03	3.00
	Tilianin	8.44E + 04	2.50E + 05	1.01	2.88
	Luteolin-7,3′-di-*O*-β-D-glucoside	1.40E + 06	4.55E + 06	1.12	3.92
	Luteolin-7-*O*-β-D-rutinoside	4.98E + 04	1.31E + 04	1.10	0.279
	Apigenin 6,8-C-diglucoside	1.44E + 05	9.00E + 00 ^c^	3.06	0.0000625
	Chrysoeriol 7-*O*-rutinoside	9.62E + 03	9.00E + 00 ^c^	2.60	0.000936
	Luteolin *O*-hexosyl-*O*-pentoside	5.68E + 04	4.80E + 03	1.53	0.0883
Flavonols	3,7-Dirhamnoside	2.47E + 05	6.10E + 04	1.15	0.251
	Sexangularetin	1.37E + 04	3.44E + 03	1.17	0.243
	Kaempferol-3,7-*O*-α-L-rhamnoside	4.18E + 04	7.32E + 03	1.24	0.202
	3,5,6,7,8,3′,4′-Heptamethoxyflavone	1.41E + 04	9.00E + 00 ^c^	2.64	0.000639
	Myricitrin	7.18E + 04	1.75E + 04	1.19	0.231
	Isorhamnetin	4.64E + 03	9.00E + 00 ^c^	2.45	0.00194
	Rhamnetin	5.46E + 04	1.72E + 04	1.06	0.314
	Epigallocatechin gallate	2.24E + 04	6.26E + 04	1.03	3.04
Flavonoid carbonosides	Isoschaftoside	4.78E + 03	9.00E + 00 ^c^	2.47	0.00188
	Isovitexin	8.62E + 03	9.00E + 00 ^c^	2.58	0.00104
	6-C-Hexosyl-apigenin *O*-feruloylhexoside	1.50E + 03	2.70E + 04	2.17	17.3
Dihydroflavonols	Taxifolin	1.16E + 05	7.13E + 05	1.33	6.12
	3-*O*-Acetylpinobanksin	2.02E + 04	5.07E + 03	1.06	0.254
Dihydroflavones	Eriodictyol	1.14E + 04	4.45E + 04	1.16	4.02
Isoflavones	Glisoflavone	9.00E + 00 ^c^	3.12E + 03	2.49	999

^a^ H, Neptune King; S, Queen. ^b^ Differentially accumulated compounds were identified using the following cutoffs: variable importance in projection (VIP) ≥ 1; fold change < 0.5 or > 2. ^c^ 9.00E + 00 represents a barely detectable level

### Transcriptome sequencing and analysis

**Fig. 3:**
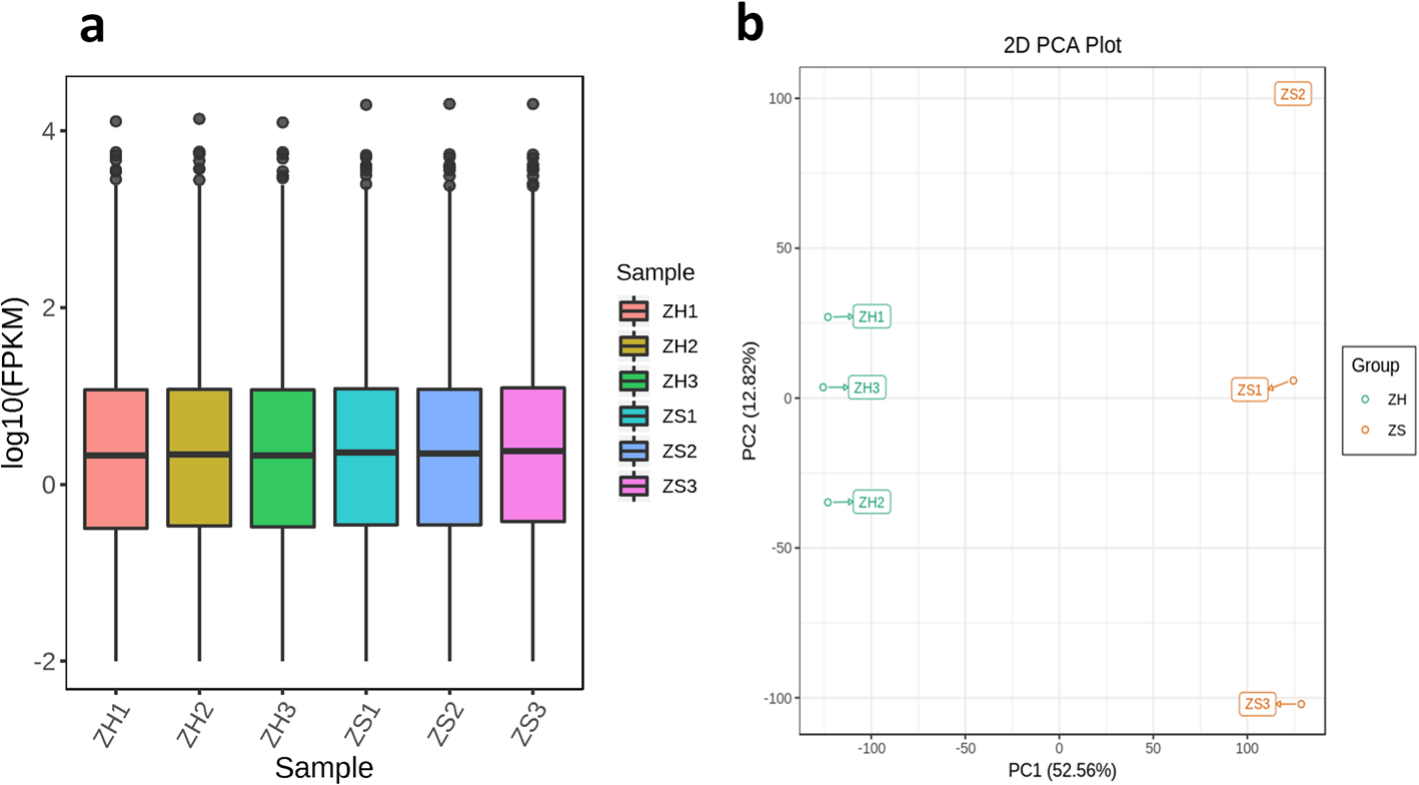
**a** Overall distribution of sample gene expression based on FPKM data; **b** Principal component analysis based on FPKM data. ZH, Neptune King genes; ZS, Queen genes

Global gene expression was further profiled in the petal samples of the two cultivars. From the six cDNA libraries (three biological replicates per sample), we obtained a total of 47.52 Gb of clean data, with more than 91% of bases scoring Q30 (Table S2, Supplementary Materials). The libraries had 46,782,302–55,533,348 clean reads, which were successfully mapped to the *Rosa chinensis* genome (https://lipm-brows-ers.toulouse.inra.fr/pub/RchiOBHm-V2/), with matching rates in the range of 80.49%–82.12%. Consequently, 5,149 novel genes were identified, 1,303 unique genes were found to be expressed in the rose petals (Table S3, Supplementary Materials). A total of 38,551 genes were detected in the rose samples and their expression levels was estimated based on the Fragments per kilobase of exon model per million reads mapped values method (Fig. 3a, Table S4, Supplementary Materials). Transcriptome based principal component analysis (PCA) of the data showed a clear distinction between H and S samples, which is similar to the results obtained based on the metabolome analysis (Fig. 3b). This implies that the DAMs in the two phenotypes were regulated by differentially expressed genes (DEGs). A total of 4,298 DEGs, including 1,851 upregulated and 2,447 downregulated genes (Figure S2, Supplementary Materials) were identified between the samples.

### Expression of genes involved in the flavonoid biosynthesis pathway

The expression patterns of genes involved in the flavonoid biosynthesis pathways were analyzed. A total of 150 genes were assigned to this pathway (Table S5, Supplementary Materials). Comparison of the H and S data showed 15 flavonoid related genes differentially expressed. All genes involved in the specific anthocyanin biosynthetic pathways, except for ANS, were upregulated in the S cultivar, which may be determinant for color-related changes (Table 2).

**Table 2:** Candidate genes related to flower color in roses

Function	Gene	Enzyme	KO ID (EC No.)	Total^a^	Up^b^	Down^c^
Anthocyanin biosynthesis	*CHS*	Chalcone synthase	K00660 (2.3.1.74)	3	3	0
	*CHI*	Chalcone isomerase	K01859 (5.5.1.6)	11	0	0
	*F3H*	Flavanone 3-hydroxylase	K00457 (1.14.11.9)	1	0	0
	*F3’H*	Flavanone 3′-hydroxylase	K05280 (1.14.13.21)	1	0	0
	*DFR*	Dihydroflavonol 4-reductase	K13082 (1.1.1.219)	5	2	0
	*ANS*	Anthocyanidin synthase	K05277 (1.14.11.19)	5	0	1
	*BZ1*	Anthocyanidin 3-*O*-glucosyltransferase	K12930 (2.4.1.115)	7	1	1
	*UGT79B1*	Anthocyanidin 3-*O*-glucoside 2′′′-*O*-xylosyltransferase	K17193 (2.4.2.51)	3	1	0
	*GT1*	Anthocyanidin 5,3-*O*-glucosyltransferase	K12938 (2.4.1.-)	5	1	0
Flavone and flavonol biosynthesis	*UGT78D2*	Flavonol 3-*O*-glucosyltransferase	K10757 (2.4.1.91)	3	1	0
	*FLS*	Flavonol synthase	K05278 (1.14.11.23)	13	3	0
	*IF7MAT*	Isoflavone 7-*O*-glucoside-6′′-*O*-malonyltransferase	K13264 (2.3.1.115)	9	1	0
Flavanone biosynthesis	*ANR*	Anthocyanidin reductase	K08695 (1.3.1.77)	2	0	0
	*LAR*	Leucoanthocyanidin reductase	K13081 (1.17.1.3)	1	0	0

^a^ The total number of transcripts analyzed. ^b^ The number of transcripts significantly upregulated in H vs. S. ^c^ The number of transcripts significantly downregulated in ZH vs. ZS. *KO* Kyoto Encyclopedia of Genes and Genomes Orthology, *EC* Enzyme Commission, *H* Neptune King, *S* Queen

### Expression patterns of transcription factors

MYB and bHLH are particular transcription factors (TF) known to control the expression levels of structural genes involved in the biosynthesis of anthocyanins [39]. Hence, we searched or the DEGs encoding TF between the two cultivars. Only 30 DEGs encoding TFs were identified. Among these TFs, we identified one bHLH (*RchiOBHmChr1g0361301*) and two MYB (*RchiOBHmChr1g0373541* and *RchiOBHmChr1g0361191*) (Table 3). *RchiOBHmChr1g0361301* (bHLH) and *RchiOBHmChr1g0361191* (MYB) were strongly up-regulated in the S cultivar, suggesting a positive regulation of anthocyanin biosynthetic structural genes*.* In contrast, *RchiOBHmChr1g0373541* (MYB) was not expressed at all in the petals of the S cultivar but was expressed in petals of the H cultivar. Comparison of the sequences of this transcript among the two cultivars did not show any nucleotide change, therefore it is probable that a mutation has occurred in the regulatory regions.

**Table 3:** Differentially expressed transcription factors between the two rose cultivars

ID	Family	H	S	log2FoldChange
*RchiOBHmChr1g0346421*	AP2/ERF-ERF	20	100	2.338
*RchiOBHmChr1g0360021*	AP2/ERF-ERF	71	278	1.973
*RchiOBHmChr1g0376641*	AP2/ERF-ERF	24,156	1740	-3.796
*RchiOBHmChr1g0376651*	AP2/ERF-ERF	797	24	-5.062
*RchiOBHmChr1g0380021*	AP2/ERF-ERF	81	27	-1.616
*RchiOBHmChr1g0361191*	bHLH	4	17	1.943
*RchiOBHmChr1g0358601*	bZIP	1004	3201	1.673
*RchiOBHmChr1g0363221*	C2C2-GATA	3990	891	-2.164
*RchiOBHmChr1g0362171*	C2H2	10	41	1.963
*RchiOBHmChr1g0370401*	C3H	47,777	21,398	-1.159
*RchiOBHmChr1g0360751*	HB-HD-ZIP	282	579	1.035
*RchiOBHmChr0c21g0500361*	HB-WOX	47	22	-1.1
*RchiOBHmChr0c31g0501441*	HB-WOX	49	23	-1.102
*RchiOBHmChr1g0365701*	HB-WOX	33	12	-1.541
*RchiOBHmChr1g0376901*	HB-WOX	453	77	-2.543
*RchiOBHmChr1g0379021*	HB-WOX	39	15	-1.353
*RchiOBHmChr1g0370681*	LOB	3	24	2.954
*RchiOBHmChr1g0318281*	MADS-MIKC	395	812	1.04
*RchiOBHmChr1g0325381*	mTERF	20	41	1.058
*RchiOBHmChr1g0361301*	MYB-related	348	1691	2.28
*RchiOBHmChr1g0373541*	MYB-related	5	0	-4.757
*RchiOBHmChr1g0360971*	NAC	10	25	1.328
*RchiOBHmChr1g0361611*	NAC	128	60	-1.082
*RchiOBHmChr1g0361661*	NAC	447	197	-1.179
*RchiOBHmChr1g0361701*	NAC	2126	587	-1.858
*RchiOBHmChr1g0361151*	Pseudo ARR-B	567	3025	2.414
*RchiOBHmChr1g0342441*	TCP	6	24	2.045
*RchiOBHmChr1g0351841*	TCP	157	433	1.464
*RchiOBHmChr1g0357671*	WRKY	3651	520	-2.817
*RchiOBHmChr1g0359091*	WRKY	5233	1494	-1.808

### Expression patterns of MATE and ABCC gene family members

It has been reported that members of the multidrug and toxic compound extrusion (MATE) and the ATP-binding cassette transporter sub-*family* C (ABCC) families play preponderant roles for the sequestration of the anthocyanins from the cytoplasm to vacuoles [40, 41]. We therefore expanded our study on the expression patterns of ABCC and MATE genes in miniature rose petals. A total of 55 and 64 ABCC and MATE genes were found expressed in the H and S samples. No ABCC gene was differentially expressed between both samples. In contrast, 10 MATE DEGs were identified, including 8 down- and 2 up-regulated MATE genes in H sample (Table S6, Supplementary Materials). The high number of down-regulated MATE genes in H samples suggests a weak sequestration of anthocyanins in vacuoles of the petals.

### qRT-PCR validation of the expression patterns of anthocyanin related genes

To further validate the RNA-seq results, nine DEGs related to anthocyanin biosynthesis were selected, and their expression levels were analyzed in H and S petals at the blooming stage using qRT-PCR. The primer sequences of the genes are shown in Table S7, Supplementary Materials. The results confirmed that anthocyanin biosynthetic and regulatory genes, including *DFR* (*RchiOBHmChr5g0037071*, *RchiOBHmChr6g0281711*), *CHI* (*RchiOBHmChr1g0372181*), *BZ1* (*RchiOBHmChr4g0393121*, *RchiOBHmChr6g0302721*), *UGT79B1* (*RchiOBHmChr5g0009571*), *MYB* (*RchiOBHmChr6g0252211*, *RchiOBHmChr7g0241861*), and *bHLH* (*RchiOBHmChr1g0361301*) were upregulated in petals of the S cultivar (Figure S3, Supplementary Materials). We noticed a complete concordance between qRT-PCR and RNA-seq results, demonstrating that the RNA-seq data and DEG analysis in this study were reliable.

### Association analysis of the metabolome and transcriptome

To demonstrate the synthetic and regulatory characteristics of the DAMs and DEGs, subnetworks were constructed to show transcript–metabolite correlations. Only pairs with a correlation coefficient > 0.8 were considered in the analysis.

**Fig. 4:**
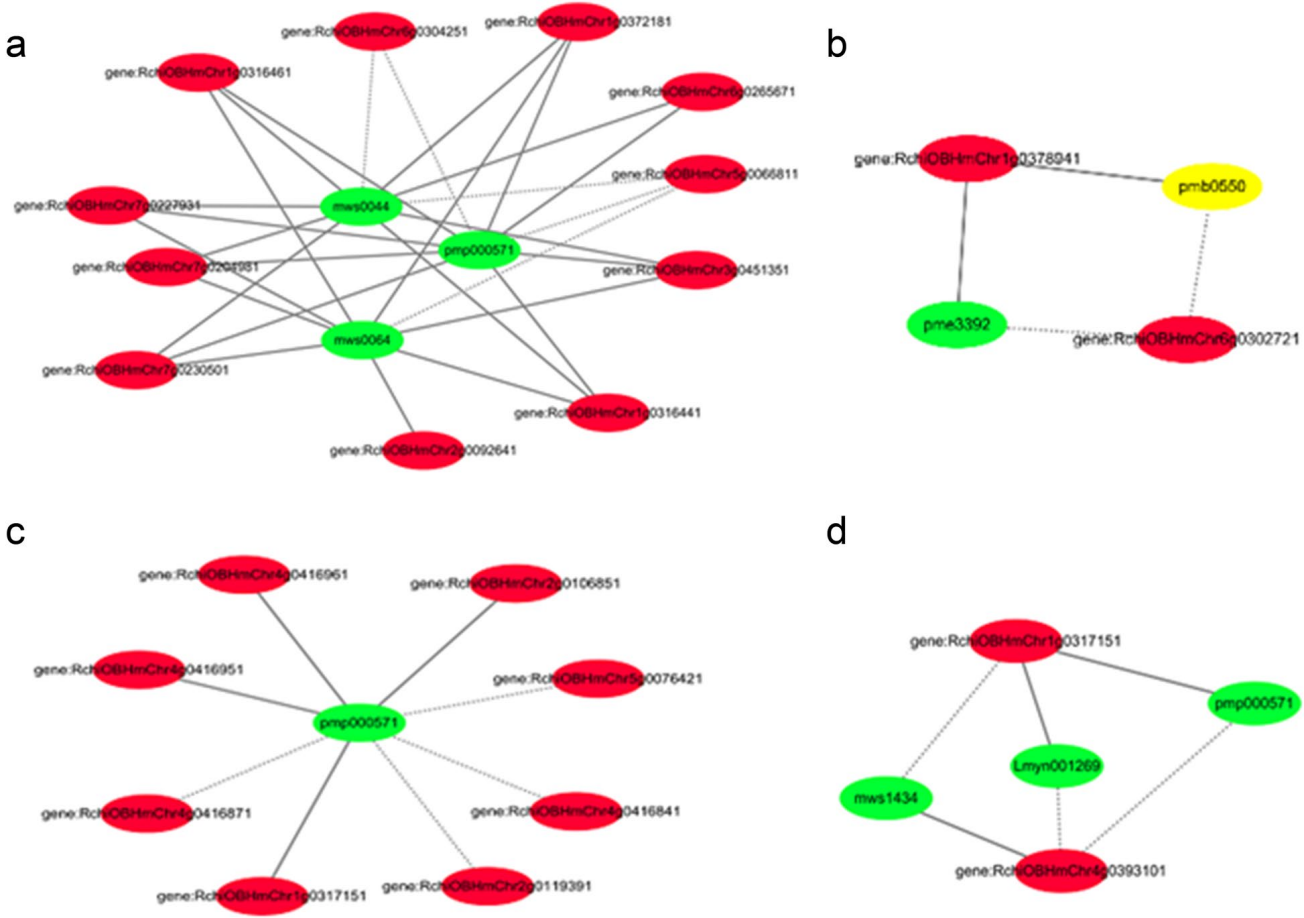
Transcript–metabolite correlation networks: **a** Ko00941; **b** Ko00942; **c** Ko00943; **d** Ko00944. Green: Metabolite; Red: gene. Solid lines: positive correlation; Dashed lines: negative correlation

Four transcript–metabolite correlation networks were constructed. Among these, Ko00941 consisted of 15 nodes and 31 edges, with 26 pairs showing a positive correlation and 5 pairs showing a negative correlation (Fig. 4a). Ko00942 consisted of five nodes and six edges, with four pairs showing a positive correlation and two pairs showing a negative correlation (Fig. 4b, Supplementary Materials). Ko00943 consisted of nine nodes and eight edges, with four pairs showing a positive correlation and four pairs showing a negative correlation (Fig. 4c). Ko00944 consisted of five nodes and six edges, with three pairs showing a positive correlation and three pairs showing a negative correlation (Fig. 4d). Detailed information on the gene–metabolite pairs involved in flavonoid biosynthesis is listed in Table S8-1–S8-4, Supplementary Materials.

These results indicate that several classic flower pigmentation-related genes were highly correlated with their corresponding metabolites, which are involved in flavonoid biosynthesis, thereby reconfirming the importance of flavonoid biosynthesis in flower color.

### Modulation of biosynthesis and accumulation of anthocyanins in rose flowers

The flavonoid pathway begins with the chalcone synthase (CHS), leading to the synthesis of naringenin chalcone from 4-coumaroyl-CoA and malonyl-CoA. Next, naringenin chalcone is isomerized by chalcone isomerase (CHI) to naringenin. Naringenin is converted into dihydrokaempferol by flavanone 3-hydroxylase (F3H). Later, dihydrokaempferol can be further hydroxylated into dihydroquercetin or dihydromyricetin by flavonoid 3′-hydroxylase (F3′H) or flavonoid 3′,5′-hydroxylase (F3′5′H), respectively. All these dihydroflavonols are converted by dihydroflavonol 4-reductase (DFR) into colorless leucoanthocyanidins and subsequently to colored anthocyanidins by anthocyanidin synthase (ANS). At the end, anthocyanidins are decorated and glycosylated by different glycosyltransferase enzymes, including, flavonoid 3-O-glucosyltransferase (UFGT) [19].

**Fig. 5:**
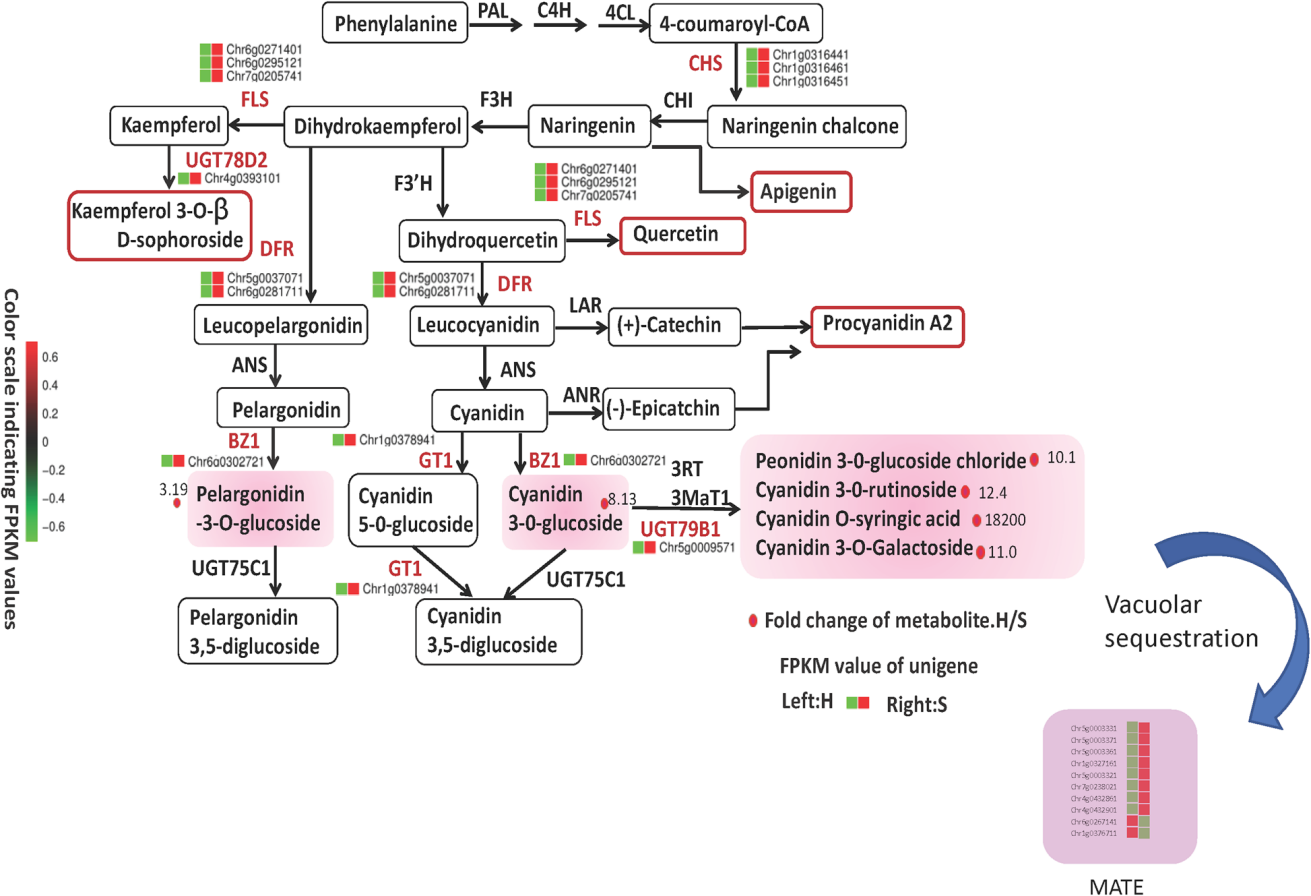
Flavonoid biosynthesis pathway and the differential metabolites of petals in miniature rose cultivar Neptune King (H) and natural mutant Queen (S). The red dots represent the up-regulated metabolites

Based on our results, we reconstructed the anthocyanin biosynthetic pathway in miniature roses. The identified anthocyanins and their relevant intermediates were rearranged to their corresponding positions (Fig. 5). The key genes involved in the anthocyanin biosynthesis were significantly upregulated in petals of the S cultivar, which was consistent with the high accumulation of anthocyanins in S. Taken together, we deduce that the coordinated upregulation of *CHS, DFR*, *FLS*, *BZ1*, *GT1, UGT78D2,* and *UGT79B1* together with the high expression levels of MATE genes play a crucial role in stimulating anthocyanin synthesis and accumulation, including cyanidin O-syringic acid, cyanidin 3-O-rutinoside, cyanidin 3-O-galactoside, cyanidin 3-O-glucoside, peonidin 3-O-glucoside chloride, and pelargonidin 3-*O*-glucoside, conferring the deep pink color in the S cultivar.

## Discussion

The aesthetic feature of flowers is of central importance in the ornamental quality and commercial value of orchids. Flower coloration has been the focus of biological studies [42–44], which have shown that there are species-specific peculiarities in the regulation of pigmentation. To contribute to the existing knowledge, species-specific studies of flower color formation and elucidation of specific regulatory mechanisms are necessary. In the present study, we investigated the underlying mechanism of color formation in two distinct flower phenotypes, the miniature rose cultivar Neptune King and its mutant, Queen rose.

### Effects of the of anthocyanins distribution on flower coloration in the two rose cultivars

A total of 190 flavonoid-related metabolites were detected and categorized into eight subgroups, including 18 anthocyanins and 11 procyanidins. This provides the most complete view of the metabolites involved in flower coloration in miniature roses. Previous studies [15–17, 45, 46] on the rose coloration process have been limited to a few flavonoid metabolites. The composition of anthocyanidins and flavonols determines the diverse phenotypes of flowers [47, 48]. The analysis of the DAMs showed that the mutant Queen was characterized by a significant high accumulation of various flavonoids. In the present study, we detected 45 differential flavonoid-related metabolites, including 7 anthocyanins. The findings indicate that cyanidin *O*-syringic acid, cyanidin 3-*O*-rutinoside, cyanidin 3-*O*-galactoside, cyanidin 3-*O*-glucoside, peonidin 3-*O*-glucoside chloride, and pelargonidin 3-*O*-glucoside accumulated to a greater extent in mutant flowers. Therefore, cyanidin derivatives can be considered the key anthocyanins responsible for the color change. It was reported that cyanidin and peonidin are responsible for orange to red colors [3, 49–54].

Anthocyanin contents have been regarded as the major factors endowing transgenic tobacco with a pink or light red flower color [55–59]. Similarly, cyanidin and peonidin have been shown to be responsible for the red color in various plants, such as tree peony, strawberry, apple, *Chrysanthemum*, *Rhododendron* and *Prunus mume* [2, 3, 60–63]. In addition, a combination of several flavonoid metabolites results in a co-pigmentation effect, which leads to deeper colors [64–66]. In the present work, the levels of one luteolin derivative, three kaempferol, three quercetin derivatives, one apigenin, were simultaneously increased in S samples, indicating a co-pigmentation effect responsible for the deep pink color observed in the mutant Queen rose.

### Major anthocyanin related genes affecting flower coloration in the two rose cultivars

Recently, high-throughput RNA-seq has been extensively used in studies on roses [67–70]. The pathways controlling flower coloration are properly characterized in plants, including roses [71, 72]. However, because of the extensive genetic variations observed in roses, varying mechanisms for color formation are expected.

In this study, the transcriptomes of Neptune King and its color mutant were analyzed, and differentially expressed flavonoid biosynthetic genes affecting the color formation were identified. The results of RNA-seq showed a significant increase in the expression of genes involved in the flavonoid biosynthetic pathway in S flowers, which strongly supported the findings of our metabolome analysis. Our findings indicated the upregulated expression of genes such as *CHS, DFR, BZ1, UGT79B1, GT1, UGT78D2, FLS,* and *IF7MAT* in S compared to H, implying that the high expression of these genes was the immediate cause for the higher accumulation of anthocyanins in mutant flowers. In previous reports, the expression of early or late biosynthetic genes, usually correlates positively with the anthocyanin content [73–80].

CHS is the primary rate-limiting enzyme that catalyzes the transformation of *p*-coumaroyl-CoA and malonyl-CoA into tetrahydroxy-chalcone in flavonoid biosynthesis. Weak expression of *CHS* restrains anthocyanin biosynthesis and leads to color changes in plant tissues. Variation of *CHS* expression levels confers color polymorphism in crabapple and arctic mustard flowers [81, 82]. In the present work, we deduce that the high expression levels of *CHS* in S flowers would provide sufficient amounts of precursor compounds for the higher anthocyanin content.

DFR is considered a critical late enzyme in anthocyanin biosynthesis. DFR catalyzes the conversion of dihydroquercetin into leucoanthocyanins which can supply flavonoids for the anthocyanin pathway. The modulation of *DFR* expression levels led to varying anthocyanins levels and pigmentations in plants such as *Dianthus caryophyllus*, *Arabidopsis*, crabapples, and rubellis [83–89].

Anthocyanidins are extremely unstable and degenerate easily; therefore, glycosylation is crucial to stabilize and transport these compounds to vacuoles, where they can function as pigments. This glycosylation role is played by *UFGT* genes [7, 90]. In the present study, *RhGT1* expression was upregulated in S flowers, which may account for the deep pink color. In accordance to our study, Griesser et al. [91] demonstrated that a downregulation of *GT1* resulted in of a significant reduction of anthocyanin pigments in ripe strawberry fruits.

MYB and bHLH are the major transcription factors regulating the expression of structural genes involved in the anthocyanin biosynthesis [39]. In this study, we identified three key genes that could be further investigated in order to facilitate the modulation of color in miniature roses.

### Downregulation of MATE genes could reduce anthocyanin sequestration in the vacuoles of H flowers

Anthocyanins, particularly the hydrophobic aglycone forms, are toxic molecules for the cell machinery due to their high chemical reactivity. Hence, after synthesis, a mechanism of anthocyanin removal is immediately triggered, leading to a sequestration in the central vacuole. High vacuolar accumulation of anthocyanins confers the observed color phenotypes in plant tissues. MATE and ABCC members have been reported as key genes involved in anthocyanins sequestrations in the vacuoles [40, 41]. Debeaujon et al. [40] were the first to discover the gene *TRANSPARENT TESTA12* (*TT12*), a MATE member, necessary for flavonoid sequestration in vacuoles of the seed coat endothelium in Arabidopsis. Later on, *TT12* has been identified, isolated and functionally validated in various plant species such as Brassicas, grapevine and blueberry [92–94]. In this study, we identified eight *TT12* upregulated in petals of the S cultivar, indicating that these genes may contribute to the high accumulation of anthocyanins observed in S.

## Conclusions

In this study, higher contents of diverse flavonoids and alterations in gene expression patterns were observed in the rose mutant as compared to its wild type. The mutation induced a significant upregulation of the key genes participating in the anthocyanin synthesis pathway, glycosylation and sequestration, resulting in significantly increased anthocyanin accumulation and darker flower coloration in the S cultivar. Functional characterization of the candidate structural genes as well as the three major transcription factors will continue.

## Methods

### Plant materials and sampling

The miniature rose cultivar Neptune King (H) and its natural mutant Queen (S) were cultivated at the Liaoning Academy of Agricultural Sciences in Shenyang City (41°48′11.75″N, 123°25′31.18″E), China. The formal identification of the plant materials was undertaken by Professor Hongmei Sun. The plants are kept at the Key Laboratory of Protected Horticulture of Education Ministry and Liaoning Province, College of Horticulture, Shenyang Agricultural University, Shenyang, China. The petal samples were collected for the metabolome study, RNA-Seq and qRT-PCR analysis. Three biological replicates were gathered per sample, with 10 blooms flowers randomly collected from 10 plants in the same batch. The petals were frozen in liquid nitrogen, and saved at − 80 °C until further use. The H and S samples used for the metabolome analysis were designated DH and DS, respectively, and those used for the RNA-seq analysis were designated ZH and ZS, respectively.

### Sample preparation and extraction

Flavonoid metabolites were extracted following a previous protocol [65], with some modifications. Petal samples were vacuum freeze-dried and ground (1.5 min at 30 Hz) to powder using a grinder. The powder (0.1 g) was weighed and dissolved in a 70% aqueous solution of methanol. To improve extraction efficiency the dissolved sample was swirled three times then stored at 4 °C overnight. The supernatant was filtered with a micropore membrane (0.22 μm pore size) after centrifugation (10,000 × *g*, 10 min), and stored in a sample injection bottle for UPLC-MS/MS analysis.

### Flavonoid identification, quantification, and data analysis

All samples were analyzed by a UPLC-MS/MS system (Shim-pack UFLC SHIMADZU CBM30A and Applied Biosystems 6500 QTRAP). A scheduled multiple reaction monitoring method was used to metabolite quantification.

Based on the self-built MWDB database (Metware Biotechnology Co., Ltd., Wuhan, China) and a public database of metabolite information, the fundamental and secondary MS records had been qualitatively analyzed using Analyst 1.6.3. The identified metabolites with significant difference in content were set with 0.5 ≥ fold change ≥ 0 or a fold change ≥ 2, *p*-value < 0.05, and VIP ≥ 1 were considered DAFs. Finally, the KEGG pathway database (http://www.kegg.jp/kegg/pathway.html) and MWDB were centered on metabolic reactions and concatenated possible metabolic pathways.

### RNA extraction, quantification, and sequencing

RNA extraction from petal samples, quantification and sequencing were conducted according to the procedures detailed by Dossa et al. [95, 96]. Six libraries representing the collected H and S petal samples (three biological replicates respectively) were prepared following standard procedures of Illumina HiSeq 4000 platform.

### Transcriptome data analysis

The raw data were cleaned by removing adaptors and low-quality reads using Fastqc with default parameters. Then, the clean reads were mapped to the *R. chinensis* genome (https://lipm-brows-ers.toulouse.inra.fr/pub/RchiOBHm-V2/) using HISAT tool. Fragments per kilobase of exon model per million reads mapped values were used for gene/transcript measurement. Differential expressed gene (DEG) analysis was conducted using the DESeq2 tool. Genes with |log_2_Fold Change|≥ 1 and a *p*-value < 0.05, were described as DEGs [96]. GO and KEGG enrichment analyses of the DEGs were conducted in the R package ‘clusterProfiler’.

### qRT-PCR analysis

Nine anthocyanin-related genes were targeted for qRT-PCR. The qRT-PCR was performed based on the same RNA samples used for sequencing [95]. The gene *Actin* was used as the internal control for transcript expression normalization. The primer pairs were designed using the Primer5 tool. The cDNAs were synthesized using SuperScript™ III First-Strand Synthesis SuperMix for qRT-PCR (Invitrogen). The qRT-PCR was conducted on an ABI 7500 Fast real-time detection system (Applied Biosystems) The reaction mix contained: 5 μl of FastStart Universal SYBR Green Master (Roche Life Sciences), 2 μl of 2 μM primer mix, 2 μl of a diluted 1:10 cDNA and ddH_2_O was added to a final volume of 10 μl. The cycling conditions were set as follow: 95 °C for 10 min, and 40 cycles of 95 °C for 15 s, and 60 °C for 1 min. The experiment was conducted with three biological replicates and three technical replicates. Data was analyzed according to the 2^−∆∆*C*t^ method [97].

## Supplementary Information

**Additional file 1: Figure S1**. (a) Multi-peak detection plot of metabolites in the multiple reaction monitoring mode; (b) Total ions current overlaps of the quality control samples by mass spectrometry detection; (c) Total ions current of one quality control sample by mass spectrometry.

**Additional file 2: Figure S2**. Differential gene volcano figure.

**Additional file 3: Figure S3**. qRT-PCR validation of the selected differentially expressed genes.

**Additional file 4: Table S1**. Types and contents of flavonoid compounds in H and S. **Table S2**: Summary of the sequencing. **Table S3**: List of novel genes identified in rose petals. **Table S4**: List of the uniquely expressed genes in rose petals. **Table S5**: A total of genes were assigned to the three pathways. **Table S6**: List of the differentially expressed MATE genes. **Table S7**: Primer sequences of genes used for qRT-PCR verification.

**Additional file 5: Table S8-1**. Gene-metabolite pairs involved in Ko00941. **S8-2**: Gene-metabolite pairs involved in Ko00942. **S8-3**: Gene-metabolite pairs involved in Ko00943. **S8-4**: Gene-metabolite pairs involved in Ko00944.

## Data Availability

The RNA-seq data has been submitted to NCBI SRA: PRJNA684357.

## Abbreviations

ANS: Anthocyanidin synthase
CHI: Chalcone isomerase
CHS: Chalcone synthase
DAF: Differentially accumulated flavonoid
DAM: Differentially accumulated metabolite
DEG: Differentially expressed gene
DFR: Dihydroflavonol 4-reductase
F3H: Flavonoid 3-hydroxylase
FLS: Flavonol synthase
GO: Gene Ontology
GT: Anthocyanidin 5,3-*O*-glucosyltransferase
KEGG: Kyoto Encyclopedia of Genes and Genomes
KOG: Eukaryotic Orthologous Groups
OPLS-DA: Orthogonal partial least squares discriminant analysis
PC1: First principal component
PCA: Principal component analysis
qRT-PCR: Quantitative real-time polymerase chain reaction
RNA-seq: RNA sequencing
UGT: UDP-glucosyltransferase
UPLC-MS/MS: Ultra-performance liquid chromatography–tandem mass spectrometry
VIP: Variable importance in projection
