# Supplementary figures and images for: Integrated metabolome and transcriptome analysis of the anthocyanin biosynthetic pathway in relation to color mutation in miniature roses
Source: BMC Plant Biol. 2021 Jun 4;21:257. doi: 10.1186/s12870-021-03063-w (PMC8176584; doi:10.1186/s12870-021-03063-w)

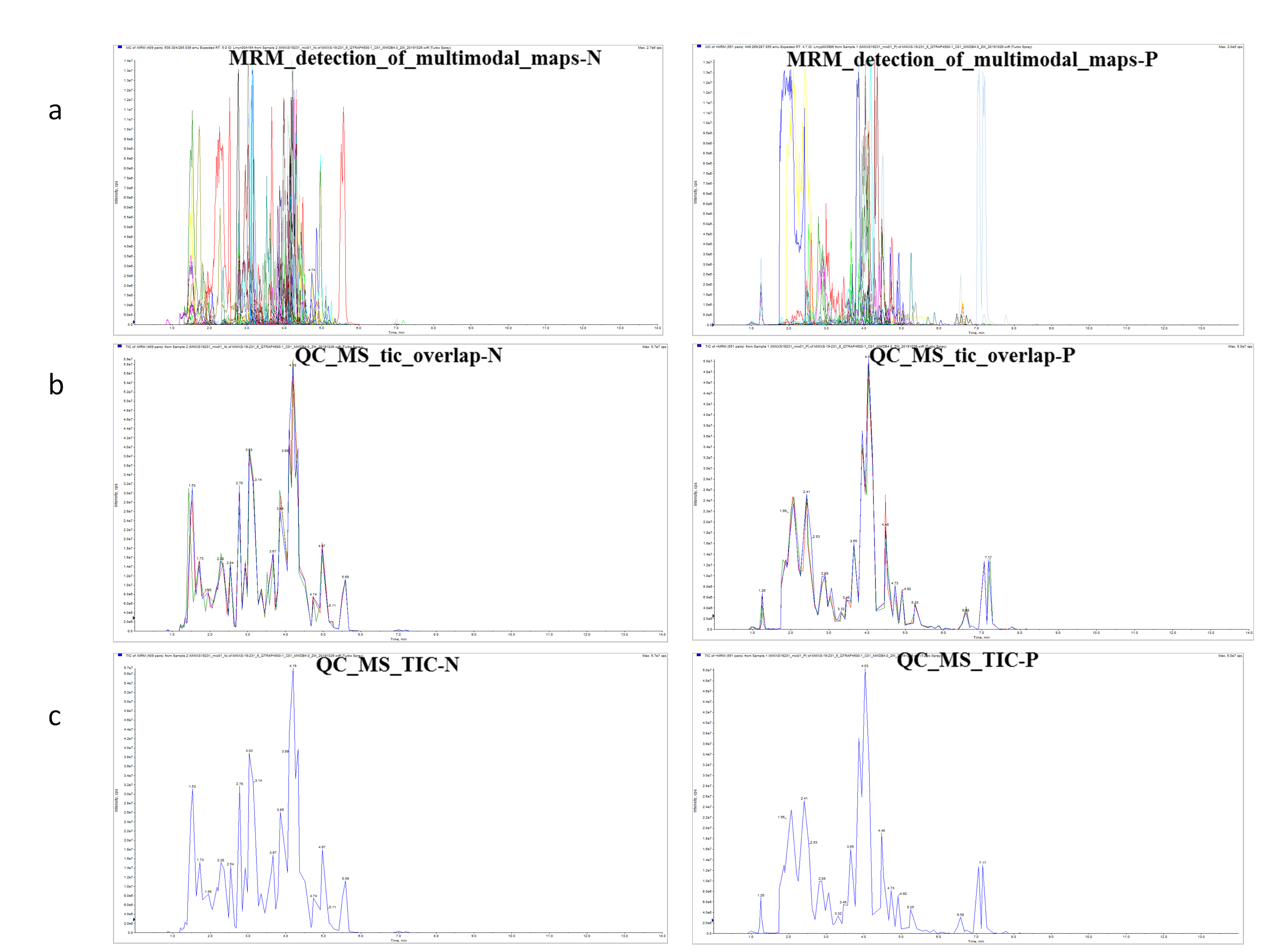

Supplement: Supplementary file 1 — Additional file 1: Figure S1. (a) Multi-peak detection plot of metabolites in the multiple reaction monitoring mode; (b) Total ions current overlaps of the quality control samples by mass spectrometry detection; (c) Total ions current of one quality control sample by mass spectrometry. [file 12870_2021_3063_MOESM1_ESM.tif]

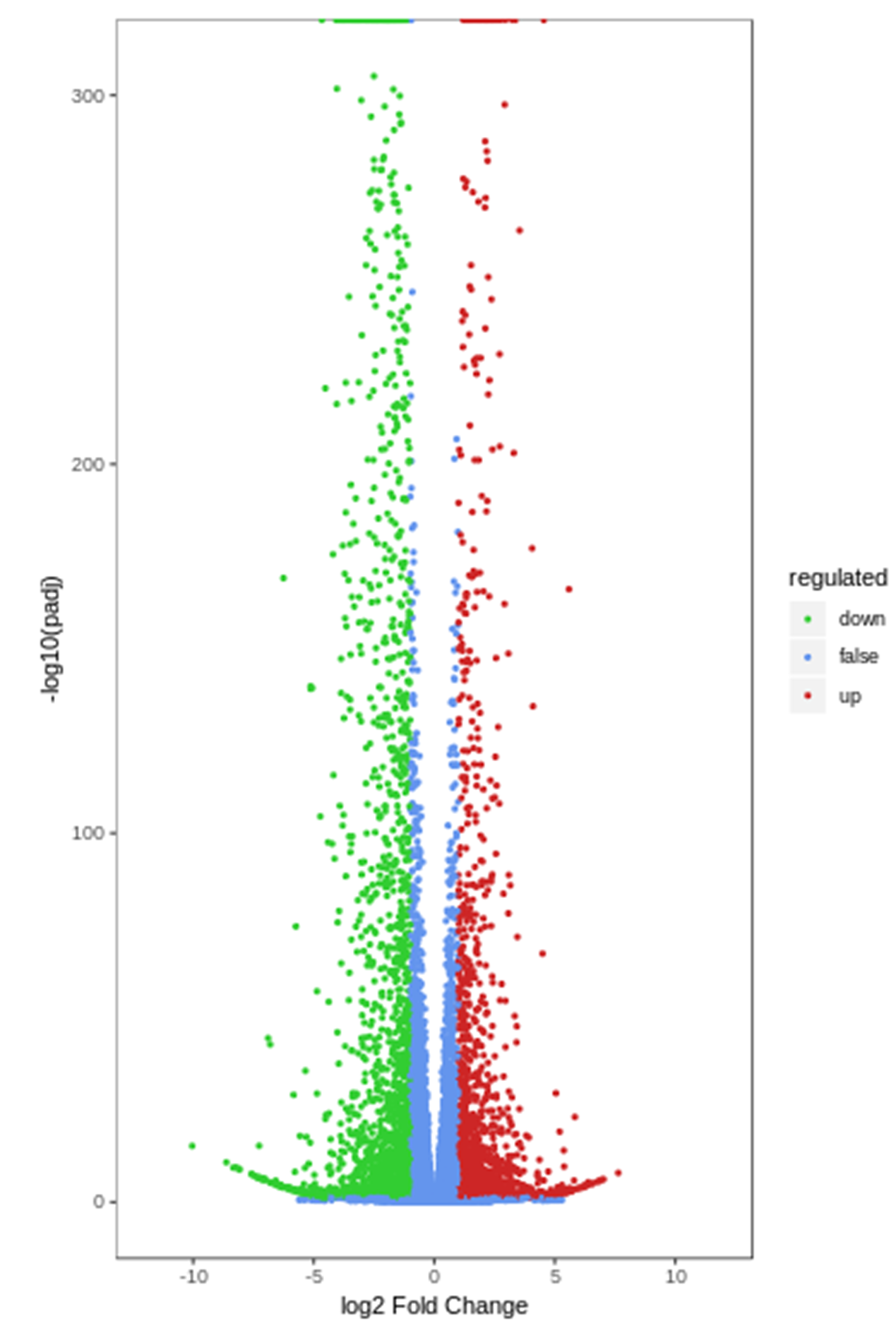

Supplement: Supplementary file 2 — Additional file 2: Figure S2. Differential gene volcano figure. [file 12870_2021_3063_MOESM2_ESM.tif]

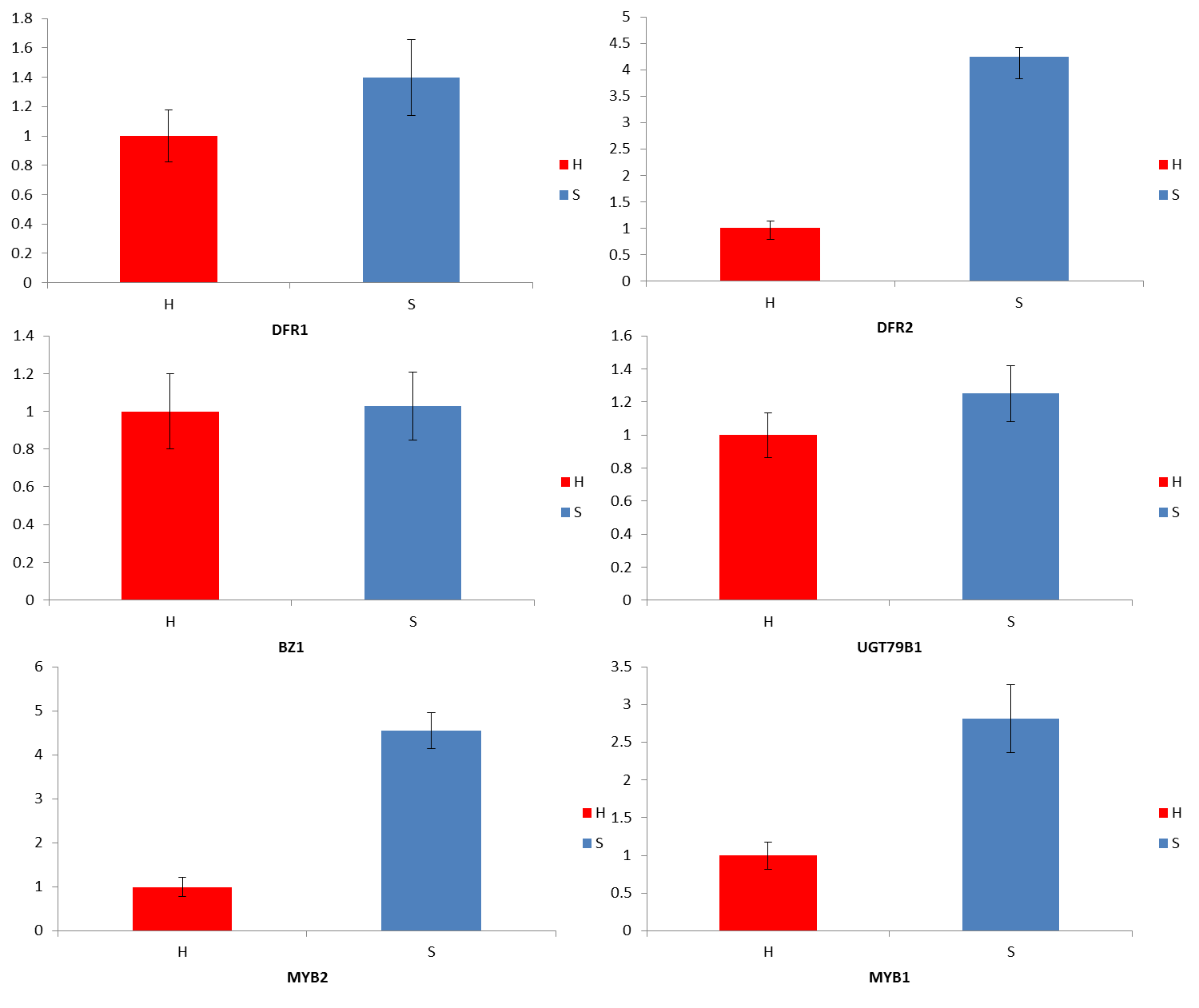

Supplement: Supplementary file 3 — Additional file 3: Figure S3. qRT-PCR validation of the selected differentially expressed genes. [file 12870_2021_3063_MOESM3_ESM.png]
